# Real-Time Fluorescence Loop Mediated Isothermal Amplification for the Diagnosis of Malaria

**DOI:** 10.1371/journal.pone.0013733

**Published:** 2010-10-29

**Authors:** Naomi W. Lucchi, Allison Demas, Jothikumar Narayanan, Deborah Sumari, Abdunoor Kabanywanyi, S. Patrick Kachur, John W. Barnwell, Venkatachalam Udhayakumar

**Affiliations:** 1 Malaria Branch, Division of Parasitic Diseases and Malaria, Center for Global Health, Centers for Disease Control and Prevention, Atlanta, Georgia, United States of America; 2 Atlanta Research and Education Foundation/VA Medical Center, Decatur, Georgia, United States of America; 3 Waterborne Disease Prevention Branch, National Center for Emerging and Zoonotic Infectious Diseases, Centers for Disease Control and Prevention, Atlanta, Georgia, United States of America; 4 Ifakara Health Institute, Dar-Es-Salaam, Tanzania; Walter and Eliza Hall Institute of Medical Research, Australia

## Abstract

**Background:**

Molecular diagnostic methods can complement existing tools to improve the diagnosis of malaria. However, they require good laboratory infrastructure thereby restricting their use to reference laboratories and research studies. Therefore, adopting molecular tools for routine use in malaria endemic countries will require simpler molecular platforms. The recently developed loop-mediated isothermal amplification (LAMP) method is relatively simple and can be improved for better use in endemic countries. In this study, we attempted to improve this method for malaria diagnosis by using a simple and portable device capable of performing both the amplification and detection (by fluorescence) of LAMP in one platform. We refer to this as the RealAmp method.

**Methodology and Significant Findings:**

Published genus-specific primers were used to test the utility of this method. DNA derived from different species of malaria parasites was used for the initial characterization. Clinical samples of *P. falciparum* were used to determine the sensitivity and specificity of this system compared to microscopy and a nested PCR method. Additionally, directly boiled parasite preparations were compared with a conventional DNA isolation method. The RealAmp method was found to be simple and allowed real-time detection of DNA amplification. The time to amplification varied but was generally less than 60 minutes. All human-infecting Plasmodium species were detected. The sensitivity and specificity of RealAmp in detecting *P. falciparum* was 96.7% and 91.7% respectively, compared to microscopy and 98.9% and 100% respectively, compared to a standard nested PCR method. In addition, this method consistently detected *P. falciparum* from directly boiled blood samples.

**Conclusion:**

This RealAmp method has great potential as a field usable molecular tool for diagnosis of malaria. This tool can provide an alternative to conventional PCR based diagnostic methods for field use in clinical and operational programs.

## Introduction

Approximately 2 billion people are exposed to malaria with morbidity surpassing 250 million cases and close to 1 million deaths per year [1]. Accurate diagnosis is critical for the proper treatment of malaria [2]. The existing tools for the diagnosis of malaria include microscopy, parasite antigen/enzyme detection kits (commonly referred to as rapid diagnostic tests (RDTs)) and molecular tools (reviewed in [3]). Each of these diagnostic tools has its own advantages and limitations. At present, microscopy and RDTs remain the only feasible options for malaria detection in many endemic countries. Microscopic diagnosis is the oldest method, can provide quantitative data and can identify species when used appropriately. Lack of infrastructure and training in most endemic countries has made microscopic diagnosis challenging which has contributed to recent interest in deploying RDTs more broadly. The current RDTs detect parasite antigens such as histidine rich protein (HRP) -2, lactate dehydrogenase (LDH) and aldolase using immunochromatographic methods. The majority of the commercial RDTs detect HRP-2 which is expressed only by *P. falciparum* but not other species and therefore this test offers specific diagnosis of *falciparum* malaria. A limitation of this test is that HRP-2 can persist in the blood for several days after the parasites are cleared therefore the assay cannot accurately tell whether someone has a current or recently treated infection. Another concern about this test is the recent discovery that up to 40% of *P. falciparum* parasites in parts of South America have deleted the *HRP-2* gene (which leads to false negative results) [4]. Most non-HRP-2 based tests (LDH and aldolase) are commonly pan-species test that allow for the speciation of *P. falciparum* and/or non-falciparum species when used in conjunction with HRP-2 based tests. Therefore, nucleic acid-based molecular methods are a potentially good alternative for malaria diagnosis as they can accurately differentiate all human-infecting *Plasmodium* species and detect low levels of parasitemia. PCR-based diagnosis recently helped to identify *P. knowlesi* in humans which had been misdiagnosed using microscopy [5]. Unfortunately, the current PCR-based methods are beyond the capacity of most malaria-endemic countries because they require sophisticated laboratory infrastructure and training which makes these techniques expensive and technically challenging to implement in simple clinical laboratories or field settings. However, as progress is made towards better malaria control and eventual goal of elimination, more sensitive diagnostic tools will be required in order to detect asymptomatic low level parasitemia. Therefore, further efforts are needed to develop next generation molecular tools for field use with a goal that such tools can complement, or in some situations, replace the existing molecular methods for malaria diagnosis and operational programs such as monitoring and evaluation of control and elimination programs.

The recently developed loop-mediated isothermal amplification (LAMP) method is a relatively simple and field-adaptable technique [6]. Parasite DNA is amplified under isothermal conditions using a polymerase with strand displacement properties (usually the *Bacillus stearothermophilus* (*Bst*) polymerase); therefore, sophisticated and expensive thermal cyclers are not required. The amplification of DNA results in the formation of magnesium pyrophosphate which appears as a precipitate as the reaction progresses. The appearance of this precipitate is used as a sign of a positive reaction. In addition, LAMP was shown to amplify DNA with high efficiency, amplifying a few copies of DNA to 10^9^ in less than 1 hour [6]. Four LAMP primers are used specific to six sites of the target sequence which makes them highly specific to the target [6]. The addition of two extra primers, known as loop-primers, was shown to accelerate the time to product formation [7], thereby shortening the required reaction time (30 minutes to 1 hr). Given that this method does not require a thermocycler or sophisticated training, it has the potential to be used as a molecular diagnostic tool for point-of-care (POC) diagnosis in both developing and developed countries provided further modifications are made. Indeed, LAMP has been used for the detection of several infectious diseases such as *Legionella* bacteria [8], West Nile Virus [9], severe acute respiratory syndrome [10], avian influenza virus [11], and norovirus [12].

Recently, the LAMP method was used for the detection of malaria parasites using the 18s rRNA gene as the target gene [13]–[17]. Poon et al. 2006 [16] reported successful application of LAMP for malaria diagnosis for the first time. They reported detecting *P. falciparum* directly from heat-treated clinical samples in which they boiled packed red blood cells at 99°C for 10 minutes, pelleted the cells by centrifugation and used the supernatant in the LAMP assay. In this study the sensitivity and specificity of LAMP was reported to be 95% and 99% respectively compared to an in-house nested PCR. In 2007, Han et al. reported a species specific LAMP diagnostic method; using clinical samples and a conventional DNA extraction method, they demonstrated sensitivity and specificity of 98.5% and 94.3% respectively compared to microscopy and a nested PCR [14]. Detection limits of 10 copies of the target 18S rRNA genes for *P. malariae* and *P. ovale* and 100 copies for *P. falciparum* and *P. vivax* were demonstrated [14]. The Plasmodium-specific and species-specific primers were shown to require less than 40 minutes for amplification. In another study, Paris et al. compared the LAMP method with both microscopy and *P. falciparum* HRP-2 RDT [13]–[17]. They found that LAMP had 100% specificity and 77.6% sensitivity when compared to HRP-2 RDT and 100% specificity and 73.1% sensitivity when compared to microscopy. However, in contrast to what was reported by Poon et al. the sensitivity and specificity of LAMP compared to a nested PCR based on primers designed by Singh et al. [18] were shown to be 79.1% and 58.3% respectively when heat treatment for DNA extraction was used [15]. Chen et al. used *P. vivax* primers to detect microscopically positive *P. vivax* clinical samples using the LAMP assay [13]. The limit of detection for *P. vivax* was shown to be 30 parasites per microliter (p/µL) [13] with 100% specificity and 98.3% sensitivity compared to microscopy.

The utility of the LAMP assay may be limited by the difficulty of visualization of precipitate especially at lower target DNA concentrations. Therefore, attempts have been made to use the intercalating dye SYBR-Green to measure the end reaction using a UV light [13], [15] or conventional real-time PCR fluorescence readers [19]–[22]. However, Paris et al. showed that the UV fluorescence method produced a high rate of false positives and suggested that this method be abandoned as a LAMP read-out [15]. Yamamura et al. combined the LAMP method with a melting curve analysis using the Genopattern Analyzer GP1000 (Yamato Scientific, Tokyo, Japan) [17]. Using clinical samples, they demonstrated LAMP sensitivity and specificity of 97.8% and 85.7% respectively, as compared to microscopy. However, the use of sophisticated equipment for diagnostic applications is not feasible in many field settings due to the lack of appropriate infrastructure. Therefore, there is a need for a simple field-usable method that can afford a quicker and objective readout for the diagnosis of malaria using the LAMP method. Here, we explored the utility of a simple portable device (tube scanner) in which both the amplification platform (heating block) and fluorescent detection unit for end point use (with the ability to acquire real time data) are combined into a single unit for LAMP assay. We refer to this method as RealAmp. We demonstrate the utility of this method for the diagnosis of malaria by using published *Plasmodium* genus specific primers and comparing it to microscopy and a nested PCR method as described by Singh et al [18].

## Methods

### Ethics Statement

Samples used in this study were obtained from a human clinical trial conducted in Tanzania to assess the efficacy of anti-malarial drugs. This study was approved by both the Ifakara Health Institute and the CDC Institutional Review Board and informed written consent forms were obtained from each subject.

### Description of the portable equipment

**Figure 1:**
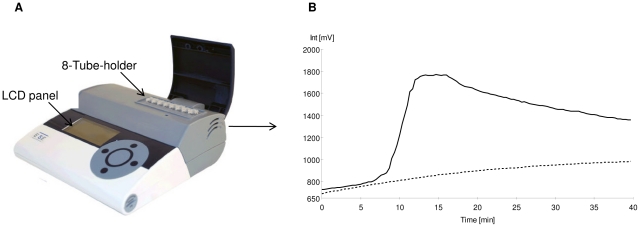
Description of the RealAmp method. The ESE-Quant Tube scanner equipped with temperature settings to amplify DNA isothermally and spectral devices to detect amplified product using fluorescence is shown (A). The tube scanner can hold 8,200 µL PCR tubes and is equipped with an LCD panel through which positive or negative results can be detected. If the tube scanner is connected to a computer with the appropriate software, the results are obtained in real-time as shown in B. The fluorescence units are shown on the Y-axis and the time to amplification on the x-axis. Amplification curves are observed (solid line) in case of a positive sample. No amplification curves (dotted line) indicate a negative sample.

The portable fluorescence reader (ESE-Quant Tube Scanner) used for this study was developed by a commercial manufacturer (ESE Gmbh, Stockach, Germany, Figure 1A). This device has an eight tube holder heating block with adjustable temperature settings and spectral devices to detect amplified product using fluorescence spectra. This equipment weighs about 2.2 lbs with the dimensions 74 mm×178 mm×188 mm (H × W × D). The unit is completely portable and can be operated with a Li-Ion rechargeable power pack without external power supply. A small LCD (monitor) is available to display the results (as positive or negative) without the need of a computer. However, the device can also be used together with a computer to generate real time amplification plots as the reaction progresses (as done in this study).

### Plasmodium parasites and clinical samples

*P. falciparum* (3D7) was cultured in our laboratory. The cultures were synchronized by the sorbitol method to select for the ring stage parasites which have single nuclei and therefore can be reproducibly used for quantitation of DNA. A thin smear was made, stained with Giemsa and the percentage parasitemia determined. The number of parasites/µL (p/µL) was determined by counting the total number of RBCs/µL using a coulter counter and using the percentage parasitemia data: (p/µL  =  RBCs/µL x percentage parasitemia). The other three human-infecting Plasmodium species, *P. vivax* (SV4), *P. malariae* (Uganda I), and *P. ovale* (Nigeria I) were acquired from infected monkeys or chimpanzees at the CDC. The parasites/µL data for these species were obtained by microscopy. To test the limits of detection of RealAmp, the DNA from these four species was diluted from 40,000 p/µL or 10,000 p/µL to 1 p/µL. In addition, *P. knowlesi* and seven other primate malaria parasites, *P. inui, P. cynomolgi, P. coatneyi P. fieldi, P. semiovale, P. fragile, P. gonderi* were tested. Ninety four samples confirmed to be *P. falciparum* positive by microscopy obtained from a human clinical trial to assess the efficacy of anti-malarial drugs and 12 samples known to be negative for *P. falciparum* (based on microscopy diagnosis) from a previous study [23] were used to assess the sensitivity and specificity of the RealAmp assay. Non-malaria infected human DNA was used as a control.

### DNA extraction

DNA was isolated from all the samples using a QIAamp DNA Mini Kit (QIAGEN, Valencia, CA-(Qiagen method)). The DNA was aliquoted and stored at −20°C. To determine the utility of the heat- treated method of DNA extraction in RealAmp cultured *P. falciparum* parasites were subjected to the heat- treatment method as described by Poon et al. [16]. Briefly, freshly cultured 3D7 parasites were adjusted to 50% hematocrit using whole blood. A starting parasitemia of 40,000 p/µL was prepared from which six 10-fold serial dilutions were prepared to a final parasite concentration of 0.4 p/µL. Fifty microliters each of these dilutions were heated on a heat-block at 99°C for 10 minutes. The tubes were then centrifuged at 13,000rpm for 3 minutes and the supernatant collected and used in the RealAmp and nested PCR assays. In parallel, an aliquot of each of these dilutions was also subjected to the Qiagen method of DNA isolation and also tested by both RealAmp and nested PCR methods.

### Nested PCR

Nested PCR was performed with primers and cycling conditions as described by Singh et al. [18] with some modifications. Reactions were performed in 20 µL total volume containing 1X buffer, 2.5 mM MgCl_2_, 200 µM dNTPs, 200 nM primers, and 1.25 units of Taq Polymerase (New England Biolabs, Ipswich, MA). The PCR amplified material was analyzed using gel electrophoresis (2% gel) to visualize the bands of appropriate size.

### RealAmp Method

The RealAmp method was performed using the commercially available Loopamp DNA amplification kit (Eiken Chemical Co., Ltd., Tokyo, Japan) following the manufacturer's instructions with the exception of the addition of 0.25 µL per 12.5 µL reaction volume of a 1∶100 diluted SYBR Green (Invitrogen) or by the use of an in-house reaction buffer. To test the utility of an in-house reaction buffer, pilot experiments were performed in a 12.5 µL total volume containing a 2X in-house buffer (40 mM Tris-HCl pH 8.8, 20 mM KCl, 16 mM MgSO_4_, 20 mM (NH_4_)SO_4_, 0.2% Tween-20, 0.8M Betaine, 2.8 mM of dNTPs each), 0.25 µL of a 1∶100 dilution SYBR green and 8 units of Bst polymerase (New England Biolabs, Ipswich, MA). Genus specific primers, as described by Han et al. [14] were used to amplify the gene coding for the 18S ribosomal RNA. DNA amplification was carried out at 63°C for 90 minutes using the ESE-Quant Tube Scanner (ESE GmbH., Stockach, Germany) which was set to collect fluorescence signals at 1 minute intervals. A typical real-time amplification plot obtained using the RealAmp method is shown in Figure 1B. In the plot, the Y-axis denotes the fluorescence units in milli-volts (mV) and the X-axis shows the time in minutes. Amplification of *P. falciparum* DNA yielded sigmoid shaped amplification curve while the control tube (no DNA) had no measurable fluorescence indicated by a flat line in the plot.

### Statistics

The sensitivity and specificity of RealAmp method was calculated using both microscopy and a nested PCR assay [18] as reference tests. The percentage specificity and sensitivity were calculated using the formulae shown below:

Sensitivity  =  true positives/(true positives + false negatives) ×100

Specificity  =  true negatives/(true negatives + false positives) ×100

In addition, 95% Confidence Intervals (95%CI) for both sensitivity and specificity were calculated.

## Results

### Detection of different Human-infecting Plasmodium species

**Figure 2:**
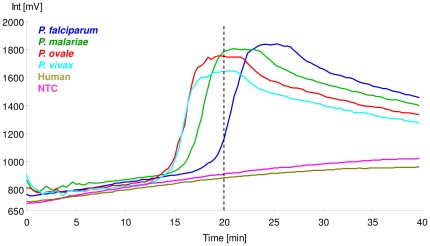
Amplification of the four human-infecting Plasmodium species using the RealAmp method. Plasmodium genus-specific primers were used to amplify the 18s ribosomal RNA gene in *P. falciparum, P. vivax, P. malariae* and *P. ovale* parasites. Amplification curves (positive) were observed for all the four species within 20 minutes (vertical dotted line). No amplification was seen with malaria-free human DNA (Human) or in the no template control (NTC).

We were able to amplify any of the four species of human malaria parasites (*P. falciparum, P. vivax, P. malariae* and *P. ovale*) within 20 minutes (Figure 2). The fluorescence peak typically persisted for about 5 minutes and then declined over time. In addition, this assay was able to detect *P. knowlesi* and seven other primate malaria parasites (*P. inui, P. cynomolgi, P. coatneyi P. fieldi, P. semiovale, P. fragile, P. gonderi* (data not shown)). No amplification as observed with the non-malaria infected human DNA control (Figure 2).

### Limits of detection of Plasmodium genus-specific RealAmp

The limits of detection of RealAmp were determined using DNA obtained from *P. falciparum, P. vivax, P. ovale and P. malariae*. The DNA was diluted from 40,000 p/µL (*P. falciparum*) or 10,000 p/µL to 1 p/µL. The limits of detection of RealAmp varied between 1–100 p/µL for the different species (Table 1). This assay required at least 1–10 p/µL for the detection of *P. ovale* and *P. malariae. P. vivax* was detected consistently at10 p/µL. For the detection of *P. falciparum* a minimum of 10–100 p/µL was required (Table 1). The nested PCR detected up to 1 p/µL for all the four species (data not shown). The time to amplification varied between 15–60 minutes. More time to amplification was required for samples with lower parasite densities although no clear correlation was observed between time to amplification and parasite densities.

**Table 1:** Detection limits of the RealAmp method tested using 10-fold serial dilutions of *P. falciparum P. vivax, P. ovale and P. malariae* DNA.

Lowest conc. detected (p/µL)	*P. falciparum*	*P. malariae*	*P. ovale*	*P. vivax*
Run # 1	10	1	1	10
Run # 2	100	10	1	10
Run # 3	10	Not tested	10	10
Run # 4	100	Not tested	Not tested	10

### Sensitivity and specificity of the RealAmp method

Clinical samples, with median parasitemia density of 3,200 p/µL (range 61–248,950 p/µL), were used to test the utility of this platform for the diagnosis of field samples. The sensitivity and specificity of the RealAmp method compared to microscopy and nested PCR is shown in Table 2. Of the 94 microscopically positive samples tested, 90 samples were confirmed to be positive by the nested PCR and 89 positive by RealAmp. Eleven out of the 12 microscopically negative samples were shown to be negative by the two methods. Overall, the sensitivity and specificity of RealAmp and nested PCR was similar when compared to microscopic data (Table 2). The RealAmp method showed 98.9% (95% CI: 93.1–99.9%) sensitivity and 100% (95% CI: 100%) specificity when compared to nested PCR.

**Table 2:** Sensitivity and Specificity of the RealAmp method and nested PCR compared to microscopy.

Microscopy (n)	Nested PCR		RealAmp	
	Positive	Negative	Positive	Negative
Positive (94)	90	4	89	5
Negative (12)	1	11	1	11
**Sensitivity**	**95.7%** (95% CI: 88.8–98.6%)		**96.7%** (95% CI: 87.5–93.8%)	
**Specificity**	**91.7%** (95% CI: 59.8–99.6%)		**91.7%** (95% CI: 59.8–99.6%)	

### Detection of Plasmodium in heat-treated samples using the RealAmp method

We compared DNA obtained by the standard Qiagen method of DNA isolation and that obtained by direct heating for their performance in RealAmp method. As shown in Table 3, the RealAmp method was able to amplify up to 40p/µL of *P. falciparum* from heat-treated samples and occasionally up to 4 p/µL whereas, up to 0.4 p/µL were detected when DNA obtained from the Qiagen method was used. There was no consistent amplification below 40 p/µL especially with heat treated DNA. No amplification was detected with heat-treated uninfected whole blood (data not shown).

**Table 3:** Amplification of *Plasmodium falciparum* from heat treated blood samples.

Amount of parasites (p/µL)	Qiagen method			Heat-Treatment method		
	Run 1	Run 2	Run 3	Run 1	Run 2	Run 3
40,000	Pos	Pos	Pos	Pos	Pos	Pos
4,000	Pos	Pos	Pos	Pos	Pos	Pos
400	Pos	Pos	Pos	Pos	Pos	Pos
40	Pos	Pos	Pos	Pos	Pos	Pos
4	ND	Pos	Pos	Pos	Pos	ND
0.4	Pos	Pos	Pos	ND	ND	ND

1 Three independent experiments (runs) are reported. Pos =  positive; ND =  not detected.

### Cost analysis of the RealAmp method compared to the nested PCR

Table 4 summarizes the cost of running the RealAmp method compared to that for nested PCR assay. The cost of performing RealAmp was lower when an in-house buffer was used. However, the cost of RealAmp increased when a commercially available buffer was used. The capital investment cost for both PCR and tube scanner was comparable (Table 4). In addition, there are important practical advantages of using RealAmp as further discussed.

**Table 4:** Cost analysis of the RealAmp method compared to the nested PCR.

	Total USD for Start- up	Total USD per sample**
Nested PCR	3,000–8000*	3.67
RealAmp using an in-house buffer	6, 344#	2.66
RealAmp using a commercial buffer	Same as above	5.05

2 *Refers to the cost of buying the equipment as listed by various major suppliers in the USA.

# Refers to price we paid for the equipment which could differ for other users.

**Cost includes all the necessary reagents and consumables; it does not include personnel cost.

## Discussion

The LAMP molecular assay is a potentially useful alternative to the current molecular tools that require sophisticated equipment and techniques [6]. A few studies have clearly demonstrated that LAMP can be used for malaria diagnosis [13]–[17]. In this study we integrated the amplification and detection stages of LAMP into one portable and simple to use platform in an attempt to make this method easily usable even in field settings. As summarized in Table 5, results from the RealAmp method were comparable to the previously reported malaria LAMP assays [13]–[17] demonstrating reasonable sensitivity and specificity profiles when compared to microscopy and nested PCR. The reported sensitivities and specificities ranged from 73.1% to 98.3% and 85.7% to 100%, respectively, using microscopy as a reference standard (Table 5). Two of these studies [15], [16] also compared malaria LAMP assays with PCR-based assays (Table 5) and one study used HRP-2 RDT as a reference [15]. The use of different reference tests, such as different PCR-based assays, clearly influences the sensitivity and specificity profile obtained. In addition, differences in the parasite densities of the samples used in the various studies may influence the sensitivities and specificities which could explain the variations observed across these studies. Out of the 94 microscopically positive samples used in this study, 4 were negative by nested PCR and 5 by RealAmp assay. The parasite density determined by microscopy for these samples ranged from 240 p/µL to 191,320 p/µL. Failure to amplify these samples by these two molecular methods was not due to low parasite density but most likely due to poor quality of the DNA preparation since we could not amplify some of the same samples using other nucleic acid tests. Further prospective studies in different transmission settings will be required to further evaluate the performance of the RealAmp method in comparison to other routine diagnostic tests.

**Table 5:** Summary of sensitivity and specificity of malaria LAMP assays reported in the literature.

Reference test	Poon et al. [16]	Paris et al. [15]	Han et al. [14]	Chen et al. [13]	Yamamura et al. [17]	RealAmp#
**Microscopy** (parasitemia)*	Not reported	Not reported	210–24,164p/µL	Not reported	0.06–6.12% parasitemia	61–248960 p/µL
Sensitivity (%)		73.1	94.3	98.3	97.8	96.7
Specificity (%)		100	98.5	100	85.7	91.7
**Nested PCR**						
Sensitivity (%)	95**	76.1/79.1**				98.8
Specificity (%)	99**	89.6/58.3**				100
**HRP-II RDT**						
Sensitivity (%)		77.6				
Specificity (%)		100				

3 *As reported by the authors.

**In these studies DNA amplification was performed using heat treated whole blood.

# Results from the current study.

One of the limitations of the current study is the fact that the RealAmp method was evaluated using only Plasmodium genus specific primers. Although the LAMP method can be used for the diagnosis of malaria parasites at the species level [14], attempts to use these published species specific primers did not yield consistent results in our hands both by conventional LAMP and RealAmp methods. We are currently evaluating the use of new DNA targets to develop primer sets that can be used for malaria species diagnosis. Despite this limitation, this genus-specific RealAmp method can be used for monitoring and evaluation of malaria control programs in the field. It can also be used as a confirmatory test for malaria infection in place of a standard PCR-based assay.

The observation that the lowest level of parasitemia required to detect *P. falciparum* was one to two orders of magnitude higher than that needed for the other three species tested can be explained by the fact that the *P. falciparum* samples used were selected for the ring stages whereas all parasite stages (rings and schizonts, the latter containing more DNA than the ring stage) were present in the other Plasmodium species. These limits of detection with this genus-specific RealAmp method are similar or better than those reported for microscopy and RDTs. Improving on the limits of detection by RealAmp (or any other malaria diagnostic test) has real potential now more than ever as there is a concerted effort to increase malaria prevention and control programs. It is hoped that these programs will lead to a reduction in malaria transmission and therefore to lower infection levels. Therefore, more sensitive tools that can be used in field settings will be needed for the evaluation of these control programs.

The RealAmp method, as reported here, was not designed for the quantitation of parasitemia. We observed that the time to amplification was shorter for samples with high parasite densities than for samples with low parasite densities. However, this relationship was noticed only when the reactions were run simultaneously: a strict correlation was not observed when samples were compared between runs indicating that one cannot draw conclusions about parasitemia levels based on the amplification time. Further efforts are needed to determine if this method can be improved for quantitative purpose.

The use of the heat-treatment method for template preparation provides a good alternative to the expensive and labor intensive DNA isolation methods that might not always be possible in field settings. In this study we were able to successfully use heat-treated samples for RealAmp amplification similar to results reported by Poon et al [16]. We did not observe any inhibition of PCR amplification as, previously reported [16], when using the heat-treated sample for DNA amplification in the nested PCR assay. The heat-treatment method yielded DNA extract that could be used to reliably detect as low as 40 p/µL. At this level of detection limit, we hope it will yield results that can be comparable to microscopic diagnosis (100–200 p/µL) in the field. This method showed slightly lower efficiency compared to Qiagen method (below 40 p/µL) and it is not clear if this difference was due to poor efficiency in DNA extraction at low parasitemia level or due to other factors. Nevertheless, these results clearly illustrate that the heat-treatment method can be further improved to make it an alternative to conventional DNA isolation methods in the field.

In our hands, the cost of running the RealAmp method was cheaper than that of the nested PCR when an in-house buffer was used in place of the commercially available buffer. The performance of our in-house buffer was as good as that of the commercial buffer and it consistently yielded similar results. Our price estimates are arbitrary and may vary for other users depending on where and how their reagents and equipment are purchased. These cost estimates do not include labor and other infrastructure costs which will vary too, depending on the region. Regardless of these cost factors, there are several important practical aspects of the RealAmp that makes it an attractive method for field use. This includes a) the fact that the tube scanner is light and small and is easily portable to even remote places while standard thermocyclers require an established laboratory setting, b) an alternate power source such as battery can be used to operate the tube scanner, c) no post-run manipulation such as gel electrophoresis is required to visualize the results contributing to shorter turnaround time, d) the RealAmp method is technically easier to perform than the nested PCR, e) this method has automation features to report results directly to remote locations, and f) this method can be modified to handle large sample numbers (e.g. using a 96-well plate holder).

The tube scanner is comparable to the real-time turbidimeter used in some studies [16] in the sense that both are capable of detecting a positive sample in real-time resulting in similar amplification plots. The tubidimeter measures the turbidity of reaction mixture while the RealAmp measures the fluorescence units generated as the product is formed. However, the tube scanner has an added feature in which the results (as positive (+) or negative (−)) can be reported on the provided LCD without the need of a computer. It would be of interest to compare the utility of these two readout machines simultaneously to determine if there is any advantage of using one over the other. We would like to point out that the RealAmp method can be performed with any alternative equipment that is similar to the tube scanner used in the study.

The utility of any diagnostic assay for point-of-care and field use will lie, among other things, on the fact that it is less expensive and simple to perform without compromising its sensitivity and specificity. The RealAmp method will be more attractive for field use if the LAMP reagents can be stored at room temperature without requiring a cold chain. Our preliminary data suggests that the RealAmp reagents can be kept at least for two weeks at room temperature without loss of activity but further studies are required to investigate this fully. In summary, this study has shown that the RealAmp method is a potential field usable tool for diagnostic purpose and for use in malaria control programs. Further field studies in different endemic countries will help to optimize its use for various malaria control applications and as a point-of-care tool.

## References

[1] WHO (2008). World Malaria Report.

[2] WHO (2010). Guidelines for the Treatment of Malaria.

[3] Bronzan RN, McMorrow ML, Kachur SP (2008). Diagnosis of malaria: challenges for clinicians in endemic and non-endemic regions.. Mol Diagn Ther.

[4] Gamboa D, Ho MF, Bendezu J, Torres K, Chiodini PL (2010). A large proportion of P. falciparum isolates in the Amazon region of Peru lack pfhrp2 and pfhrp3: implications for malaria rapid diagnostic tests.. PLoS ONE.

[5] Cox-Singh J, Davis TM, Lee KS, Shamsul SS, Matusop A (2008). Plasmodium knowlesi malaria in humans is widely distributed and potentially life threatening.. Clin Infect Dis.

[6] Notomi T, Okayama H, Masubuchi H, Yonekawa T, Watanabe K (2000). Loop-mediated isothermal amplification of DNA.. Nucleic Acids Res.

[7] Nagamine K, Hase T, Notomi T (2002). Accelerated reaction by loop-mediated isothermal amplification using loop primers.. Mol Cell Probes.

[8] Annaka T (2003). [Rapid and simple detection of Legionella species by LAMP, a mew DNA amplification method].. Rinsho Biseibutshu Jinsoku Shindan Kenkyukai Shi.

[9] Parida M, Posadas G, Inoue S, Hasebe F, Morita K (2004). Real-time reverse transcription loop-mediated isothermal amplification for rapid detection of West Nile virus.. J Clin Microbiol.

[10] Hong TC, Mai QL, Cuong DV, Parida M, Minekawa H (2004). Development and evaluation of a novel loop-mediated isothermal amplification method for rapid detection of severe acute respiratory syndrome coronavirus.. J Clin Microbiol.

[11] Imai M, Ninomiya A, Minekawa H, Notomi T, Ishizaki T (2006). Development of H5-RT-LAMP (loop-mediated isothermal amplification) system for rapid diagnosis of H5 avian influenza virus infection.. Vaccine.

[12] Fukuda S, Takao S, Kuwayama M, Shimazu Y, Miyazaki K (2006). Rapid detection of norovirus from fecal specimens by real-time reverse transcription-loop-mediated isothermal amplification assay.. J Clin Microbiol.

[13] Zheng C, Xie P, Chen Y (2002). Recombinant Mycobacterium bovis BCG producing the circumsporozoite protein of Plasmodium falciparum FCC-1/HN strain induces strong immune responses in BALB/c mice.. Parasitol Int.

[14] Han ET, Watanabe R, Sattabongkot J, Khuntirat B, Sirichaisinthop J (2007). Detection of four Plasmodium species by genus- and species-specific loop-mediated isothermal amplification for clinical diagnosis.. J Clin Microbiol.

[15] Paris DH, Imwong M, Faiz AM, Hasan M, Yunus EB (2007). Loop-mediated isothermal PCR (LAMP) for the diagnosis of falciparum malaria.. Am J Trop Med Hyg.

[16] Poon LL, Wong BW, Ma EH, Chan KH, Chow LM (2006). Sensitive and inexpensive molecular test for falciparum malaria: detecting Plasmodium falciparum DNA directly from heat-treated blood by loop-mediated isothermal amplification.. Clin Chem.

[17] Yamamura M, Makimura K, Ota Y (2009). Evaluation of a new rapid molecular diagnostic system for Plasmodium falciparum combined with DNA filter paper, loop-mediated isothermal amplification, and melting curve analysis.. Jpn J Infect Dis.

[18] Singh B, Bobogare A, Cox-Singh J, Snounou G, Abdullah MS (1999). A genus- and species-specific nested polymerase chain reaction malaria detection assay for epidemiologic studies.. Am J Trop Med Hyg.

[19] Lin X, Chen Y, Lu Y, Yan J (2009). Application of a loop-mediated isothermal amplification method for the detection of pathogenic Leptospira.. Diagn Microbiol Infect Dis.

[20] Njiru ZK, Mikosza AS, Armstrong T, Enyaru JC, Ndung'u JM (2008). Loop-Mediated Isothermal Amplification (LAMP) Method for Rapid Detection of Trypanosoma brucei rhodesiense.. PLoS Negl Trop Dis.

[21] Njiru ZK, Ouma JO, Enyaru JC, Dargantes AP (2010). Loop-mediated Isothermal Amplification (LAMP) test for detection of Trypanosoma evansi strain B.. Exp Parasitol.

[22] Varga A, James D (2006). Use of reverse transcription loop-mediated isothermal amplification for the detection of Plum pox virus.. J Virol Methods.

[23] Jain V, Armah HB, Tongren JE, Ned RM, Wilson NO (2008). Plasma IP-10, apoptotic and angiogenic factors associated with fatal cerebral malaria in India.. Malar J.

